# Relationship between Skin Temperature Variation and Muscle Damage Markers after a Marathon Performed in a Hot Environmental Condition

**DOI:** 10.3390/life11080725

**Published:** 2021-07-21

**Authors:** Daniel Rojas-Valverde, Randall Gutiérrez-Vargas, Braulio Sánchez-Ureña, Juan Carlos Gutiérrez-Vargas, Jose I. Priego-Quesada

**Affiliations:** 1 Clínica de Lesiones Deportivas (Rehab&Readapt), Escuela Ciencias del Movimiento Humano y Calidad de Vida (CIEMHCAVI), Universidad Nacional de Costa Rica, Heredia 86-3000, Costa Rica; 2 Centro de Investigación y Diagnóstico en Salud y Deporte (CIDISAD), Escuela Ciencias del Movimiento Humano y Calidad de Vida (CIEMHCAVI), Universidad Nacional de Costa Rica, Heredia 86-3000, Costa Rica; rangutie@live.com; 3 Núcleo de Estudios en Alto Rendimiento Deportivo y Salud (NARS), Escuela Ciencias del Movimiento Humano y Calidad de Vida (CIEMHCAVI), Universidad Nacional de Costa Rica, Heredia 86-3000, Costa Rica; bsanchez@una.cr (B.S.-U.); jucagu@msn.com (J.C.G.-V.); 4 Programa de Ciencias del Ejercicio y la Salud (PROCESA), Escuela Ciencias del Movimiento Humano y Calidad de Vida (CIEMHCAVI), Universidad Nacional de Costa Rica, Heredia 86-3000, Costa Rica; 5 Centro de Estudios para el Desarrollo y Rehabilitación en Salud (CEDERSA), Escuela Ciencias del Movimiento Humano y Calidad de Vida (CIEMHCAVI), Universidad Nacional de Costa Rica, Heredia 86-3000, Costa Rica; 6 Research Group in Sports Biomechanics (GIBD), Department of Physical Education and Sports, University of Valencia, 46010 Valencia, Spain; 7 Biophysics and Medical Physics Group, Department of Physiology, University of Valencia, 46010 Valencia, Spain

**Keywords:** endurance, infrared thermography, thermal image, recovery, creatine kinase

## Abstract

This study aimed to assess the effect of a marathon running at a hot environmental temperature on the baseline skin temperature (Tsk) of the posterior day and to analyze the relationship between Tsk response and muscle damage markers variation. The Tsk, creatine kinase, and lactate dehydrogenase of 16 marathon runners were assessed four times before (15 days and 45 min) and after (24 h and 6 days) a marathon in a hot environment (thermal stress index = 28.3 ± 3.3 °C and humidity ~81%). The Tsk of thirteen different body regions of both right and left lower limbs were analyzed. Higher values after the marathon were observed than 45 min before in creatine kinase (174.3 ± 136.4 UI/L < 1159.7 ± 699.7 UI/L, *p* < 0.01 and large effect size) and lactate dehydrogenase (362.6 ± 99.9 UI/L < 438 ± 115.5 UI/L, *p* = 0.02 and moderate effect size). Generally, Tsk was higher the day after the marathon than at the other three moments (e.g., rectus femoris region, 6 days before vs. the day after, 95% confidence interval of the difference (0.3, 1.6 °C), *p* = 0.04 and large effect size). No relationship or correlation was observed between the variation of Tsk and muscle damage markers (*p* > 0.05). In conclusion, performing a marathon in a hot environmental condition results in a higher Tsk the day after the marathon. This increase in Tsk could be because of the heat generated by the marathon and its subsequent physiological processes (e.g., increase in endothelial nitric oxide, glycogen resynthesis, or increase of systemic hormones), which would be reflected in the Tsk due to the peripheral vasodilation promoted by the hot environment. However, among these processes, muscle damage does not seem to be of great importance due to the lack of an observed relationship between Tsk and muscle damage markers.

## 1. Introduction

Endurance competition such as marathon running is recognized for having a high physical and physiological demand [1]. Marathon running could lead to muscle damage due to the cumulus of the high volume of concentric–eccentric muscle contractions [2]. Moreover, muscle fibers damage may provoke leakage of the muscle proteins into the bloodstream and later to subsequent complications, including but not limited to delayed onset muscle soreness (DOMS), rhabdomyolysis, and acute kidney injury [2,3,4]. After a marathon, monitoring muscle damage is vital to improve posterior recovery and training schedules and reduce injury risk [5,6].

Muscle damage induced by exercise is commonly assessed by quantifying blood markers such as serum creatine kinase (sCK), serum lactate dehydrogenase (sLDH), myoglobin, magnesium, among others [7,8,9,10]. Although sCK and sLDH are considered good indicators of muscle damage [11], other indirect tools have been used to decrease the invasive procedure during blood collection, such as using visual analog scales to measure DOMS [12]. Although the monitorization of baseline skin temperature (Tsk) using infrared thermography (IRT) was another methodology proposed, the results of the different investigations are contradictory [13,14,15,16,17].

The Tsk assessment to obtain information about muscle damage is based on its relationship with inflammation and skin blood flow alterations [16,18,19]. It was suggested that inflammation resulting from muscle damage could increase muscle temperature and alter Tsk [17,19]. However, a recent study indicated that muscle damage increases peripheral vasoconstriction, covering up inflammatory effects [16]. In addition, while some studies observed increments of baseline Tsk in the posterior days after exercise [14,17], others did not find differences [13,15,16], which makes further investigation more necessary. Finally, most of these studies performed the exercise in moderate environmental conditions, such as a marathon at 16 °C [16], a half-marathon at 15 °C [15], calf-rising repetitions at 23 °C [13], or triathlon training camp at 12 °C [14], and only one study in which the exercise was soccer matches at higher temperatures (28 °C) [17].

Because the possible effect of environmental temperature during exercise could explain the contradictory results observed by the studies, a study with high environmental temperature and a very physiologically demanding activity such as a marathon could improve the understanding of this topic. A high environmental temperature could enhance skin blood flow [20] and reduce the intensity of skin blood vasoconstriction after the competition, increasing the heat transfer between the muscle and the skin.

Therefore, the objective of this study was to assess the effect of a marathon running performed at a hot environmental temperature on baseline Tsk of the following day and to analyze the relationship between baseline Tsk response and muscle damage marker variations (sCK and sLDH). It was hypothesized that the high thermal stress caused by the long duration exercise in a high environmental temperature could result in skin temperature increments 24 h after the marathon and have a better relationship with the muscle damage markers than previous studies.

## 2. Materials and Methods

### 2.1. Design

**Figure 1:**
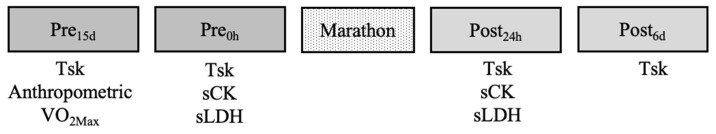
Study design of assessment of skin temperature (Tsk), anthropometric characteristics (body mass, height, body fat percentage, and lean mass), maximum oxygen uptake (VO_2Max_), and blood biomarkers: serum creatine kinase (sCK) and serum lactate dehydrogenase (sLDH), in four testing days (Pre_15d_: 15 days before the marathon; Pre_0h_: 45 min before the marathon; Post_24h_: 24 h after marathon; and Post_6d_: 6 days after the marathon.

This study was a crossover experimental design with the aim of exploring the relationship between Tsk and biochemical (sCK and sLDH) responses using data obtained previously and after the Tamarindo Beach Marathon, Costa Rica (altimetry = 0–80 m, outdoor wet-bulb globe temperature (WGBT), also known as thermal stress index = 28.3 ± 3.3 °C and humidity ~81%). Four different days of testing were performed: two tests before and two tests after running the marathon, at baseline conditions. The pre-event measurements were 15 days before (Pre_15d_) and 45 min before the event (Pre_0h_) without warm-up, and 24 h (Post_24h_) and 6 days (Post_6d_) post-marathon (see Figure 1). As Pre_0h_ could be affected by a greater activation of the sympathetic activity of the autonomic nervous system to be a measurement close to the competition [15], Tsk was assessed at Pre_15d_ to have a more realistic baseline value. Moreover, Tsk was also measured at Post_6d_ to assess thermal recovery (if Tsk was increased by the competition) and whether it reached values similar to Pre_15d_, also to be understood as a baseline measure to compare the Post_24h_ Tsk values.

### 2.2. Participants

Participants were recruited after contacting running clubs. Sixteen recreational marathon runners volunteered to participate in the study (9 males and 7 females: age 36 ± 7 years, body mass 66.8 ± 12.0 kg, height 167 ± 11 cm, fat percentage 21 ± 8%, maximum oxygen consumption 53.4 ± 7.0 mL/kg/min, total lean mass 48.9 ± 11.3 kg, finish time 4 h 07 min ± 35 min). Age, maximum oxygen consumption, and finish time were not different between sex (*p* > 0.05), but females presented lower body mass, height, and total lean mass and higher fat percentage (*p* < 0.01). Inclusion criteria were to have previous experience running marathons (3 ± 3 marathons before the event and 10 ± 7 years of running experience) and being already registered in the event before researchers contact. Exclusion criteria were to suffer neuromuscular injury at least 3 months before the event and any pathological or metabolic disease. All participants signed an informed consent based on the Declaration of Helsinki. The protocol was approved by the MSc Committee of the National University (RegN°01-2016).

### 2.3. Procedures

#### 2.3.1. Runners’ Characterization

Participants were characterized by anthropometric characteristics and maximum oxygen uptake (VO_2_max) 15 days before the marathon. Body mass was assessed using a digital scale (sensitivity of 0.1 kg) (Elite Series BC554, Tanita-Ironman^®^, Chicago, IL, USA), and height was measured using a stadiometer (SECA, Hamburg, Germany). Body composition (body fat percentage and lean mass) was obtained through a dual x-ray absorptiometry (DEXA) with an error of ± 3% (General Electric in CORE 2011^®^, Milwaukee, WI, USA).

VO_2_max was assessed through an incremental test with intensity increases every 2 min until volitional fatigue using a gas analyzer (VO_2000_, MedGraphics^®^, Saint Paul, MN, USA) with an accuracy of ±3% of absolute volume [21]. Data analysis was performed using BreezeSuite^®^ software.

#### 2.3.2. Skin Temperature

Lower limb Tsk was assessed using an IRT camera (T440, FLIR Systems, Wilsonville, OR, USA). The camera had a focal plane size of 320 × 240 pixels with a measurement uncertainty of ±2% and a thermal sensitivity of 0.04 °C. A specific checklist to measure human Tsk [22] was followed to ensure the quality of the measurements. Day-to-day thermograms were taken at the same hour of the day (7:00–7:30 a.m.) to avoid circadian body temperature changes [23]. Before the evaluation, the athlete was asked to avoid intense workload (e.g., <1 h, <85% of maximum heart rate) at least 24 h before measurements [24] except for the evaluation 24 h after the day of the event. Participants were also requested to avoid lotions, creams, or any other topical substance. Alcohol and caffeine consumption was forbidden at least four hours before evaluations.

Thirty minutes before each assessment, the camera was turned on. The camera was located using a tripod three meters from participants at the height of 60 cm with a 5° angle to reduce any potential light reflection [25]. To standardize assessments, the images were taken by fixing the center point of the image on the center between participants' knees, which was checked using a camera-incorporated laser. An anti-reflective panel was used as the background of the thermogram to ensure the uniformity of the images. In addition, reflecting or heat/cold emissions were avoided to prevent undesired infrared radiation.

After lower limb skin was cleaned up with water and then dried, thermal images were taken after participants remained in an anatomical position for 15 min, wearing underwear and without moving or touching their skin, in a thermo-neutral room (23.0 ± 0.5 °C and relative humidity of 58 ± 6%, WGBT, 3R) controlled by an air-conditioning system. This procedure aimed to standardize Tsk as a thermal acclimation to achieve equilibrium [26].

**Figure 2:**
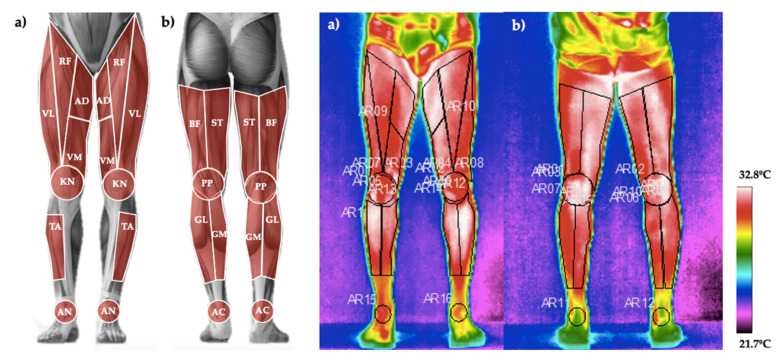
(**a**). Regions of interest measured in the anterior plane: rectus femoris (RF), vastus lateralis (VL), vastus medialis (VM), adductor (AD), knee (KN), tibialis anterior (TA) and ankle (AN). (**b**). Regions of interest measured in the posterior plane: biceps femoris (BF), semi-tendinous (ST), popliteus (PP), gastrocnemius lateralis (GL), gastrocnemius medialis (GM) and Achilles (AC).

Thirteen different regions of interest (ROIs) of the posterior and anterior plane of both right and left lower limbs were measured (total of twenty-six; Figure 2) using a thermographic software (ThermaCAM^®^, Researcher^TM^ Pro 2.10, FLIR Systems, Wilsonville, OR, USA). From each ROI, the mean value of the Tsk registered in all pixels was obtained. Emissivity was set at 0.98 [27]. Dominance was determinate by participants’ self-report (all participants were right-leg dominants). The selected ROIs were similar to other previous studies [28] as follows (see Figure 2): rectus femoris, vastus lateralis, vastus medialis, adductor, knee, biceps femoris, popliteus, semi-tendinous, gastrocnemius lateralis, gastrocnemius medialis, ankle, anterior tibialis, and Achilles. Additionally, the Delta (ΔTsk) was calculated as the difference between the Post_24h_ and the Pre_15d_. Pre_0h_ was not used due to the possible effect of the activation of the sympathetic activity of the autonomic nervous system before the competition, as suggested by a previous study [15].

#### 2.3.3. Serum Test

Blood was extracted 45 min before and 24 h after the event from the antecubital vein using a 5 mL blood collection sterile tube (BD Vacutainer^®^, New York, NY, USA). The tube contained a spray-coated silica particles activator and gel polymer to facilitate serum separation during centrifugation. Samples were centrifuged for 10 min at 2000× *g* relative centrifugal force using a tube centrifuge (PLC-01, Gemmy Industrial Corp., Taipei, China). During the data collection stage, blood samples were stored on ice in a special cooler (45QW Elite, Pelican^TM^, Torrance, CA, USA) until serum samples were frozen at −20 °C (~5 hours after blood extraction). Sample analysis and processing were performed 24 h after data collection in an isolated and temperature-controlled laboratory using a semi-automatic biochemical analyzer (RT-1904C, Rayto^®^, Shenzhen, China) by photometry method. The variables analyzed as muscle damage indicators were sCK and sLDH (both in UI/L units) [7,8,9,10]. All procedures were performed under relevant protocols for the handling and disposal of biological materials, according to the manufacturer's instructions for the equipment and reagents used. For blood markers, the Delta percentage (ΔCK and ΔLDH) was calculated as the difference between the Post_24h_ and the Pre_0h_, divided by Pre_0h_.

### 2.4. Statistical Analysis

Statistical analysis was performed using RStudio software (version 1.2.5033). The significance level was set at 0.05 for all analyses, and results are expressed as means ± standard deviation (SD). The normality of the data of each of the variables was verified by the Shapiro–Wilk test (*p* > 0.05), except for the muscle damage variables (*p* < 0.05). For each ROI, repeated-measures two-way ANOVA with time factor (Pre_15d_ vs. Pre_0h_ vs. Post_24h_ vs. Post_6d_) and dominance factor (dominant vs. non-dominant lower limb) was applied. The post hoc analysis was performed by Bonferroni's method when significance was found in the ANOVA models. The difference between Pre_0h_ and Post_24h_ in sCK and sLDH was analyzed using Wilcoxon tests. For significant pair differences of parametric analysis, 95% confidence interval of the differences (95%CI) are provided, and Cohen's effect sizes (ESd) were computed and classified as follows: 0.2–0.4 *small*; 0.5–0.7 *moderate* and >0.8 *large* [29]. For nonparametrical data, Rosenthal’s r was calculated (ESr) and classified as follows: 0.1–0.2 *small*; 0.3–0.4 *moderate* and >0.5 *large* [30].

To analyze the associations between ΔTsk, muscle damage, and participant characterization, stepwise multiple linear regressions were performed with ΔTsk as predicting variables. The inputs of the models were: age, sex, fat percentage, number of marathons completed in the past, running experience, time performed in the marathon, VO_2_max, ΔCK, and ΔLDH. Final models were then adjusted to retain only variables yielding *p*-values <0.05. For the models obtained, the coefficient of each variable of the equation, the percentage of the variance explained by the model (*R^2^*), and the significance value of the model was provided. Moreover, bivariate Pearson correlation analysis was performed between ΔTsk, ΔCK, and ΔLDH. To reduce error type I, the number of ΔTsk was simplified by performing a principal component analysis (PCA) with varimax rotation of the orthogonal rotation method [31]. Therefore, the ΔTsk of the regions grouped by PCA were averaged.

## 3. Results

Higher values after the marathon were observed in sCK (174.3 ± 136.4 UI/L (Pre_0h_) < 1159.7 ± 699.7 UI/L (Post2_4h_), *p* < 0.01 and *ESr* = 0.8 (*large*)) and sLDH (362.6 ± 99.9 UI/L (Pre_0h_) < 438 ± 115.5 UI/L (Post2_4h_), *p* = 0.02 and *ESr* = 0.3 (*moderate*)).

**Figure 3:**
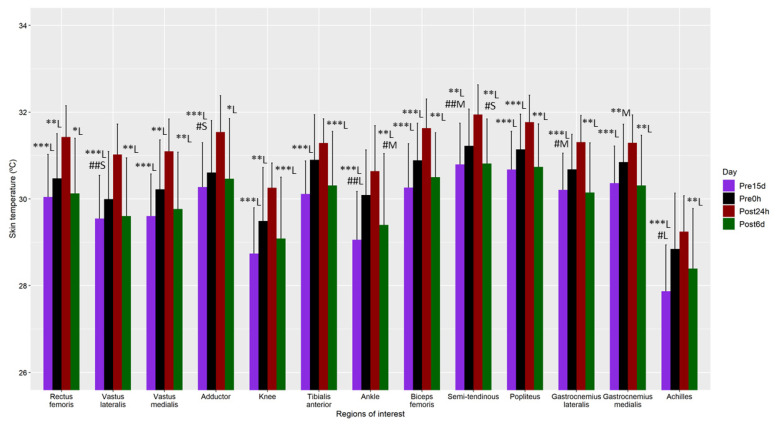
Mean and standard deviation of the skin temperature measurements 15 days before (Pre_15d_) and 45 min before the marathon (Pre_0h_), and 24 h (Post_24h_) and 6 days (Post_6d_) post marathon. Differences are shown using symbols (diff with Post_24h_: * *p* < 0.05, ** *p* < 0.01, *** *p* < 0.001; diff with Pre_0h_: # *p* < 0.05, ## *p* < 0.01), and the Cohen’s effect size by letter (S—small effect size, M—moderate effect size, L—large effect size).

Regarding the Tsk assessment, first, the main effect of dominance was not significant in any of the ANOVAs (*p* > 0.05), nor was its interaction with the time factor (*p* > 0.05). Therefore, this factor was not considered in the following results. The main effect of the time factor was significant for all the ROIs (*p* < 0.05). Generally, Tsk was higher at Post_24h_ than at the other three moments (e.g., rectus femoris: Post_24_ vs. Pre_15d_ 95%CI (0.9, 2.0°C), *p* < 0.001 and *ESd* = 1.6 (*large*); difference Post_24_ vs. Pre_0h_ 95%CI (0.4, 1.4 °C), *p* < 0.01 and *ESd* = 1.1 (*large*); difference Post_24_ vs. Post_6d_ 95%CI (0.3, 1.6 °C), *p* = 0.04 and *ESd* = 1.3 (*large*)) (Figure 3).

Moreover, Tsk was also higher at Pre_0h_ than at Pre_15d_ or Post_6d_ for vastus lateralis (difference vs. Pre_15d_ 95%CI (0.5, 1.7 °C), *p* < 0.01 and *ESd* = 0.4 (*small*)), adductor (difference vs. Pre_15d_ 95%CI (0.4, 1.6 °C), *p* = 0.02 and *ESd* = 0.3 (*small*)), ankle (difference vs. Pre_15d_ 95%CI (0.5, 1.7 °C), *p* < 0.01 and *ESd* = 1.0 (*large*); difference vs. Post_6d_ 95%CI (0.3, 1.6 °C), *p* = 0.02 and *ESd* = 0.5 (*moderate*)), semitendinous (difference vs. Pre_15d_ 95%CI (0.5, 1.8 °C), *p* < 0.01 and *ESd* = 0.5 (*moderate*); difference vs. Post_6d_ 95%CI (0.4, 1.7 °C), *p* = 0.01 and *ESd* = 0.4 (*small*)*),* gastrocnemius lateralis (difference vs. Pre_15d_ 95%CI (0.3, 1.5 °C), *p* = 0.02 and *ESd* = 0.6 (*moderate*)) and Achilles (difference vs. Pre_15d_ 95%CI (0.2, 1.4 °C), *p* = 0.04 and *ESd* = 0.8 (*large*)). No differences were observed between Pre_15d_ and Post_6d_. (*p* > 0.05).

PCA analysis suggested a three-component structure (PCs), explaining a total of 90% of the variance. PC1 explained 54% of the variance and was composed of the ROIs of the posterior lower limb: biceps femoris, semi-tendinous, popliteus, gastrocnemius lateralis, gastrocnemius medialis, and Achilles. PC2 explained the 21% of the variance and was composed of the ROIs of the anterior thigh (rectus femoris, vastus lateralis, vastus medialis, adductor) and knee. Finally, PC3 explained the 15% of the variance and was composed of the ROIs of the anterior leg (tibialis anterior and ankle).

Regression analysis (Table 1) showed that ΔTsk of the posterior lower limb presented a positive relationship with running experience and an inverse relationship with age. Women showed higher values for ΔTsk of the posterior lower limb and ΔTsk of the anterior thigh. ΔCK and ΔLDH were not included in any model and were not correlated with any of the ΔTsk variables (*p* > 0.21).

## 4. Discussion

The purpose of this study was to analyze the effect of a marathon run performed at a hot environmental temperature on baseline Tsk and its relationship with muscle damage markers. It was hypothesized that the high thermal stress caused by a marathon in a high environmental temperature could result in Tsk increments 24 h after the marathon and could have a direct relationship with muscle damage markers. The main results of this study partially confirmed the hypothesis because Tsk 24 h after the marathon was higher than the other measurement days; however, no relationship was observed between Tsk and muscle damage markers.

The marathon itself entails significant physiological challenges, such as depletion of energy resources, dehydration, hyponatremia, hyperthermia, oxidative stress, transient insulin resistance, immune system depression, structure damage, and decreased functional capacity [10,32]. Regarding Tsk, the present study showed an increment of Tsk the day after a marathon, which is the opposite of a previous study that did not show an alteration in skin temperature 24 and 48 h after a marathon [16]. The main difference between the two studies, explaining the discrepancy between the results, is the mean environmental temperature where the marathon was performed. While the mean environmental temperature of the mentioned study was 16 °C, the present study was carried out in a hotter environmental scenario (28.3 ± 3.3 °C and humidity ~81%). In this sense, after physical exercise, the body performs a series of physiological responses to maintain body homeostasis [7,8,10]. This challenge to maintain internal stability is increased in the presence of hot and humid conditions [10,32].

Some previous studies have proposed IRT as an effective tool for evaluating the muscle damage resulting from exercise [17,19]. However, in recent years, numerous studies have not shown changes in Tsk in the days after performing an exercise with muscle damage, and no relationships were observed between Tsk and muscle damage markers [13,15,16,33]. Some of these studies have suggested that a possible explanation is that although there is an increase in muscle temperature, the muscle damage or pain could be producing peripheral vasoconstriction that does not allow this effect to be reflected on Tsk [15,16]. The higher Tsk observed the day after a marathon in the present study could suggest that a hot environment facilitates peripheral vasodilation so that increases in muscle temperature are reflected in the Tsk. Although it could be assumed that in this caseTsk is related to inflammation and muscular ruptures, no relationship was observed between the variation of Tsk and muscle damage markers. The increase in internal or muscular temperature may be not only due to muscle damage but also to other processes, such as the increase in endothelial nitric oxide, glycogen resynthesis, or increase of systemic hormones [34,35,36], explaining this lack of correlations. Therefore, although the current results suggest that IRT can be proposed as an effective tool for assessing the effect of exercise on the posterior days in a hot environmental scenario, more evidence is needed to support this idea.

A positive methodological aspect of the present study was that Tsk was measured 15 days before the marathon and 6 days after to have a control Tsk pattern. This was done because a previous study suggested that the Tsk the day before a competition may be higher due to a greater activation of the sympathetic activity of the autonomic nervous system [15]. In this sense, first, no differences were observed between the measurements 15 days before and 6 days after, suggesting that these Tsk data could be considered control values as had been hypothesized. Second, the Tsk on the day of the marathon was higher in some regions than these control values, which could be due to greater activation of the sympathetic activity [15,37], as has been commented.

Regression models showed some relationships between the variation of Tsk and other participant's characteristics (age, running experience, and sex), which should be considered with caution due to the sample size of the present study, and therefore should be considered in future lines of research. Age was inversely related to the variation of Tsk, which could be explained by the reduction of capacity of peripheral vasodilation/vasoconstriction experienced with increasing age [38,39]. The positive relationship observed with running experience can be related to physical fitness level. Trained people usually have a greater capacity for heat transference between the core and the skin due to higher peripheral vasodilation capacity [40]. Finally, women showed higher values of Tsk variation, which is the opposite of what some literature suggested, as women usually have a lower capacity of Tsk response due to a higher body fat percentage [41,42]. In this sense, our only explanation may be that it is affected by the menstrual cycle [43,44], something that is complete speculation since this information was not asked of the participants, which is a limitation of the study.

The present results have critical practical applications since they add information to the scientific evidence on the use of IRT to evaluate the effects of physical exercise. In this sense, the results suggest that the environmental temperature to which people are subjected during the day influences these effects and that assessing in hot environmental conditions can improve said analysis. However, future studies are necessary to corroborate these ideas, evaluating the impact of different exercise intensities carried out in hot environmental conditions on the temperature of the following day or evaluating the effect of a marathon on the same participants under two different weather conditions.

While the results of this study have provided information regarding the potential use of IRT to assess the effect of exercise on Tsk, some limitations to the study are present. One of the limitations of this study concerns the sample; it would be interesting to extend this research to include more participants to assess factors such as sex or physical fitness level properly. Another limitation of the study is that other physiological parameters that may have helped to interpret the results were not measured, such as core and muscle temperature or skin blood flow. In this sense, applying a cold stress test could be valuable for obtaining more information on the alteration of the peripheral vasodilation/vasoconstriction capacity [16]. Finally, a control group that did not perform exercise would increase the validation of the results obtained.

## 5. Conclusions

Performing a marathon in a hot environmental condition results in a higher Tsk the day after the marathon. This increase in Tsk could be because of the heat generated by the marathon and its subsequent physiological processes (e.g., increase in endothelial nitric oxide, glycogen resynthesis, or increase of systemic hormones), which would be reflected in the Tsk due to the peripheral vasodilation promoted by the hot environment. However, among these processes, muscle damage does not seem to be of great importance because no relationship was observed between Tsk and muscle damage markers.

## Figures and Tables

**Table 1:** Regression models obtained by multivariate stepwise regression analyses using as predicting variables variations in skin temperature (ΔTsk) and as inputs: age, sex, fat percentage, number of marathons performed in the past, running experience, time performed in the marathon, VO2max, ΔCK, and ΔLDH.

	Regression Models Obtained		
Predicting Variable	Variable	Coefficient (CI95%)	*R^2^* (*p*-Value)
ΔTsk Posterior lower limb	Constant Sex * Age Running experience	1.79 (0.66, 2.93) 1.28 (0.73, 1.82) −0.05 (−0.09, −0.01) 0.07 (0.03, 0.11)	0.71 (<0.01)
ΔTsk Anterior thigh and knee	Constant Sex *	1.14 (0.80, 1.47) 0.66 (0.16, 1.17)	0.36 (0.01)
ΔTsk Anterior leg	No variable was included		

* Note: For sex, men had a value of 1 and women of 2.
